# Plasma cytokine profiling in sibling pairs discordant for autism spectrum disorder

**DOI:** 10.1186/1742-2094-10-38

**Published:** 2013-03-14

**Authors:** Valerio Napolioni, Benjamin Ober-Reynolds, Szabolcs Szelinger, Jason J Corneveaux, Traci Pawlowski, Sharman Ober-Reynolds, Janet Kirwan, Antonio M Persico, Raun D Melmed, David W Craig, Christopher J Smith, Matthew J Huentelman

**Affiliations:** 1 Neurogenomics Division, The Translational Genomics Research Institute (TGen), 445 N Fifth Street, Phoenix, AZ, 85004, USA; 2 Child Neuropsychiatry Unit - Laboratory of Molecular Psychiatry and Neurogenetics, University Campus Bio-Medico, Rome, Italy; 3 Laboratory of Molecular Psychiatry and Psychiatric Genetics, Department of Experimental Neurosciences, I.R.C.C.S. Fondazione Santa Lucia, Rome, Italy; 4 Center for Rare Childhood Disorders, The Translational Genomics Research Institute, Phoenix, AZ, USA; 5 The Southwest Autism Research and Resource Center (SARRC), Phoenix, AZ, USA; 6 Current address: Clinical Molecular Lab, Clarient, Aliso Viejo, CA, USA

## Abstract

**Objective:**

Converging lines of evidence point to the existence of immune dysfunction in autism spectrum disorder (ASD), which could directly affect several key neurodevelopmental processes. Previous studies have shown higher cytokine levels in patients with autism compared with matched controls or subjects with other developmental disorders. In the current study, we used plasma-cytokine profiling for 25 discordant sibling pairs to evaluate whether these alterations occur within families with ASD.

**Methods:**

Plasma-cytokine profiling was conducted using an array-based multiplex sandwich ELISA for simultaneous quantitative measurement of 40 unique targets. We also analyzed the correlations between cytokine levels and clinically relevant quantitative traits (Vineland Adaptive Behavior Scale in Autism (VABS) composite score, Social Responsiveness Scale (SRS) total T score, head circumference, and full intelligence quotient (IQ)). In addition, because of the high phenotypic heterogeneity of ASD, we defined four subgroups of subjects (those who were non-verbal, those with gastrointestinal issues, those with regressive autism, and those with a history of allergies), which encompass common and/or recurrent endophenotypes in ASD, and tested the cytokine levels in each group.

**Results:**

None of the measured parameters showed significant differences between children with ASD and their related typically developing siblings. However, specific target levels did correlate with quantitative clinical traits, and these were significantly different when the ASD subgroups were analyzed. It is notable that these differences seem to be attributable to a predisposing immunogenetic background, as no other significant differences were noticed between discordant sibling pairs. Interleukin-1β appears to be the cytokine most involved in quantitative traits and clinical subgroups of ASD.

**Conclusions:**

In the present study, we found a lack of significant differences in plasma-cytokine levels between children with ASD and in their related non-autistic siblings. Thus, our results support the evidence that the immune profiles of children with autism do not differ from their typically developing siblings. However, the significant association of cytokine levels with the quantitative traits and the clinical subgroups analyzed suggests that altered immune responses may affect core feature of ASD.

## Introduction

Autism spectrum disorders (ASDs) are a heterogeneous group of severe neurodevelopmental disorders characterized by atypical social interactions, impaired communication, and tendency to engage in idiosyncratic, repetitive, or restrictive behaviors, with onset before 3 years of age. ASDs include autistic disorder, Asperger’s syndrome, and pervasive developmental disorder-not otherwise specified (PDD-NOS) [1]. Although significant progress has been made in the identification of genes and copy-number variants associated with syndromic autism (approximately 10% of the total number of cases with ASD) [2], little is currently known about the etiology of idiopathic non-syndromic autism. The clinical heterogeneity of ASD probably reflects the complexity of its genetic underpinnings, involving multiple contributing loci, genetic heterogeneity, epistasis, and gene-environment interactions [3].

In addition to the results from neurobiological research in ASD, highlighting the pathways involved in neural development, synapse plasticity, structural brain abnormalities, cognition, and behavior, converging evidence point to the existence of altered immune function in ASD, which directly affects some or all these neurological processes [4]. Several immune abnormalities have been reported in ASD, including familial autoimmune disorder clustering and ASDs [5], altered gene expression, resulting in dysfunctional natural killer (NK) cells [6], immune transcriptome alterations in the temporal cortex of subjects with autism [7], and the presence of auto-antibodies to the cerebellum in children with autism [8]. Indeed, children with ASD were reported to have higher levels of auto-antibodies (including anti-myelin basic protein [9], anti-myelin-associated glycoprotein [10], anti-ganglioside [11], anti-neuronal [12], and anti-mitochondrial [13] antibodies) compared with healthy children. Despite the extensive research linking immune irregularities to ASDs, there are no salient findings that have significantly advanced the understanding of the pathogenesis of ASD.

Previous studies [14-25] have reported altered cytokine levels in subjects with autism with inconclusive results, perhaps attributable to different types of study design, but also probably reflecting the wide heterogeneity of ASD. In addition, one study found no difference in cytokine levels between young ASD children and normotypic controls [26]. Moreover, relatively few cytokines have been examined to date, and recent technologies have opened higher throughput means for quantitatively surveying 10s to 100s of unique cytokines per sample on an array. Previous studies have shown that patients with autism have higher cytokine levels in cases of autism compared with controls or subjects with other developmental disorders [14-25]. In the current study, we evaluated whether these alterations occur within families with ASD by performing a comprehensive plasma-cytokine profiling in 25 sibling pairs discordant for ASD. We also analyzed the correlations between cytokine levels and clinically relevant quantitative traits (Vineland Adaptive Behavior Scale in Autism (VABS) composite score, Social Responsiveness Scale (SRS) total T score, head circumference, and full intelligence quotient (IQ)). In addition, because of the high phenotypic heterogeneity of ASD, we divided the patients into four defined subgroups (those who were non-verbal, those with gastrointestinal (GI) issues, those with regressive autism, and those with a history of allergies), which encompass common and/or recurrent endophenotypes in ASD, and tested the cytokine levels of these groups [27-30].

## Materials and methods

### Ethics approval

The study protocol was approved by the Western Institutional Review Board (WIRB; number 20071224). All parents received a comprehensive description of the study, and gave written informed consent for their children’s participation.

### Subjects

The study, entitled ‘DNA, RNA, and Proteomics Case–control Study of Individuals with Autism’, was carried out in collaboration with the Translational Genomics Research Institute (TGen, Phoenix, AZ, U.S.A.). Recruitment was carried out by the Southwest Autism Research and Resource Center (SARRC) (Phoenix, AZ, USA) The study targeted whole families in which at least one child had a clinical diagnosis of autism according to the criteria of the DSM-IV (*Diagnostic and Statistical Diagnostic and Statistical Manual of Mental Disorder*s, Fourth Revision) [1]. To minimize confounding genetic factors, only male discordant sibling pairs younger than 15 years of age were selected for cytokine analysis. In total, 25 sibling pairs were enrolled.

Briefly, the psychiatric, medical, and family histories of all participants were obtained, and the participants underwent behavioral, sensory, and cognitive questionnaires and assessments. All the recruited subjects were free of any active treatment with pharmacological or other agents. To further characterize the disorder in each proband, the Autism Diagnostic Observation Schedule (ADOS) [31] and the Autism Diagnostic Interview-Revised (ADI-R) [32] were used by research reliable raters. Adaptive functioning was assessed using the VABS, behavior impairments were determined using the SRS [33], and the IQ was evaluated using the Stanford-Binet Intelligence Scales (fifth edition). Head circumference (occipital frontal circumference) was measured using a flexible non-stretchable measuring tape graded in millimeters.

### Cytokine analysis

Peripheral blood samples were collected from each subject for plasma analysis before any experimentation was carried out. Whole blood was collected into EDTA-coated collection tubes (K_2_EDTA Vacutainer; BD, Franklin Lakes, NJ, USA). The tube was inverted 10 times to mix the blood with the EDTA, and stored on ice until further processing. Each tube was processed within 4 hours of collection. In all cases, the duration from collection to freezing was noted. Blood was separated by centrifugation at 1,000 × g in a swinging bucket centrifuge pre-chilled to 4°C. The plasma was harvested, divided into 200 μl aliquots, and stored at −80°C until cytokine analysis (no freeze-thaw cycles occurred before analysis).

Cytokine analysis was conducted using an array-based multiplex sandwich ELISA system (Quantibody® Human Inflammation Arrays; QAH-INF-3, RayBiotech, Inc. Norcross, GA, USA) for simultaneous quantitative measurement of 40 unique inflammatory cytokines/chemokines (B-lymphocyte chemoattractant (BLC; also known as CXCL13), eotaxin, eotaxin-2, granulocyte colony-stimulating factor (G-CSF), granulocyte–macrophage colony-stimulating factor (GM-CSF), I-309, intercellular adhesion molecule (ICAM)-1, interferon (IFN)-γ, interleukin (IL)-1α, IL-1β, IL1 receptor antagonist (IL-1ra), IL-2, IL-4, IL-5, IL-6 and its soluble rceptor (IL-6sR), IL-7, IL-8, IL-10, IL-11, IL-12p40, IL-12p70, IL-13, IL-15, IL-16, IL-17, monocyte chemotactic protein (MCP)-1, macrophage colony-stimulating factor (M-CSF), monokine induced by gamma interferon (MIG), macrophage inflammatory protein (MIP)-1α, MIP-1β, MIP-1δ, platelet-derived growth factor (PDGF)-BB, regulated on activation normal t cell expressed and secreted (RANTES), tissue inhibitor of metalloproteinase (TIMP)-1, TIMP-2, tumor necrosis factor (TNF)-α, TNF-β, and soluble receptors TNF-sRI and TNF-sRII).

Samples were tested according to the manufacturer’s instructions. Briefly, one array was run to optimize the plasma dilutions at which the majority of cytokines would be quantified within the array’s limit of detection (LOD). All plasma samples were diluted 1:1 using sample diluent, then 50 μl of each of diluted samples and prepared standards were incubated on the arrays at 4°C overnight with gentle shaking. After several washes, the detection antibody cocktail was added and the arrays incubated at room temperature for 2 hours with gentle shaking. After another series of washes, streptavidin conjugated to a Cy3 equivalent dye was added, and the arrays incubated for 1 hour with gentle shaking. After a final washing step, the arrays were imaged using a microarray scanner (Agilent Microarray Scanner; Agilent, Santa Clara, CA, USA) and the fluorescence data was extracted using the accompanying software (Agilent Feature Extraction software; Agilent). Raw fluorescence data was analyzed using the Q-Analyzer software for the QAH-INF-3 arrays (RayBiotech, Inc., Norcross, GA, USA), which was used to calculate cytokine concentrations in pg/ml based on a seven-point linear regression of the standard curves.

### Statistical analysis

The quantitative data were not normally distributed, as assessed by Kolmogorov linear regression, and are thus presented as median ± interquartile range (IQR; that is, 25th to 75th percentile) or semi-interquartile range (IQR/2), and contrasted using non-parametric statistics. Differences between discordant sibling pairs were evaluated by the Wilcoxon rank sum test. The Mann–Whitney test was used to compare variables between unpaired groups. Correlations between cytokine levels and quantitative clinical variables were performed by Spearman’s rank correlation (ρ). Statistical significance was set at *P*<0.05. Nominal *P* values are presented, because the cytokine levels were non-independent variables and, under similar conditions, correction for multiple testing remains controversial [34,35]. All analyses were performed using the SPSS statistics software, (version 18.0; SPSS Inc., Chicago, IL, USA).

## Results

### Subjects

The demographic and clinical characteristics of all participants are summarized in Table 1. There was no significant difference in age distribution between children with ASD and their healthy siblings (*z* = 1.232, *P* = 0.218). As expected, all VABS and SRS scores were significantly different between the two groups (Table 1). The subjects with ASD group had a significantly lower total IQ than did the healthy sibling group (*z* = −2.201, *P* = 0.028). Head circumference was not significantly different between subjects with ASD and the healthy siblings (*z* = −0.805, *P* = 0.421); however, using the Moses Test for extreme reactions, significant differences in range between the two groups were noticed (*P* = 0.040). There was a higher incidence of macrocephaly (that is, occipitofrontal circumference >97th percentile or +2 SD) in the group with ASD than in their related siblings (30.0% vs. 23.8%). Notably, this percentage is in the range previously reported for the ASD population [36].

**Table 1:** Demographic and clinical characteristics of study participants

	**Children with ASD, n = 25**	**Healthy siblings, n = 25**	***Z *score**	***P *value**
Ethnicity, n (%)				
Caucasian	21 (88.0)		–	–
Hispanic	1 (4.0)		–	–
Caucasian-Hispanic	1 (4.0)		–	–
Asian	1 (4.0)		–	–
Age, years, mean ± SD	8.11 ± 3.65		7.44 ± 3.12	1.232	0.218
VABS scores, median ± IQR/2					
Communication	54.5 ± 14.3		100.0 ± 10.5	−3.945	**3.9 × 10**^**-5**^
Daily living skills	55.5 ± 12.8		95.0 ± 11.5	−3.875	**5.3 × 10**^**-5**^
Motor skills	75.0 ± 12.5		103.0 ± 8.5	−3.180	**7.4 × 10**^**-4**^
Composite	53.5 ± 10.8		94.0 ± 11.0	−3.920	**4.4 × 10**^**-5**^
SRS scores, mean ± SD					
Social awareness	75.3 ± 11.0		46.8 ± 10.3	3.516	**2.2 × 10**^**-4**^
Social cognition	80.8 ± 11.4		46.2 ± 7.7	3.516	**2.2 × 10**^**-4**^
Social communication	82.9 ± 11.7		47.1 ± 13.1	3.516	**2.2 × 10**^**-4**^
Social motivation	76.6 ± 10.7		49.8 ± 15.8	3.258	**5.6 × 10**^**-4**^
Mannerisms	85.8 ± 12.6		49.1 ± 14.0	3.516	**2.2 × 10**^**-4**^
T score, total	85.8 ± 11.8		47.3 ± 12.8	3.516	**2.2 × 10**^**-4**^
Head circumference, median percentile ± IQR/2	82.0 ± 16.0		84.0 ± 10.0	−0.805	0.421
Full IQ, median ± IQR/2	71.0 ± 12.0		105.5 ± 10.0	−2.201	**0.028**
Subgroups, n (%)					
Non-verbal	7 (28.0)		NA	–	–
GI issues	7 (28.0)		NA	–	–
Regression	8 (32.0)		NA	–	–
Allergy history	5 (20.0)		NA	–	–

Abbreviations: GI, gastrointestinal; IQR/2, semi-interquartile range; NA, not assessed; SD, standard deviation. Significant differences are shown in bold.

The percentage of children with ASD who were non-verbal (defined as the complete absence of intelligible words at time of diagnostic assessment of autism) was 28.0%. The same percentage was found for children with ASD displaying GI issues (presenting at least one of the following symptoms: 1) constipation, 2) diarrhea, 3) abdominal bloating, discomfort, or irritability, 4) gastroesophageal reflux or vomiting, 5) feeding issues or food selectivity). Regressive autism (when a child appears to develop typically but then starts to lose speech and social skills, typically between the ages of 15 and 30 months, and is subsequently diagnosed with autism [28]) was seen in 32% of the study group, while 20% had a history of allergies.

### Cytokine profiles of discordant sibling pairs

The differences in cytokine/chemokine levels between discordant sibling pairs were analyzed (see Additional file 1: Table S1). None of the measured parameters showed significant differences between children with ASD and their related healthy siblings. Further, we performed a *post hoc* analysis to detect possible changes in T helper (Th)1 (IL-2 + IFN-γ + TNF-α), Th2 (IL-4 + IL-5 + IL-6 + IL-10 + IL-13), and Th17 (IL-6 + IL-17) cytokine levels and their related ratios, but no significant differences were detected (data not shown).

### Correlations between cytokine levels and quantitative traits

Correlations between cytokine levels and clinically relevant quantitative traits (VABS composite score, SRS total T score, head circumference, and full IQ) were performed for the 25 children with ASD, using Spearman’s ρ. We also evaluated correlations between the cytokines and the quantitative trait being examined (see Additional file 2: Table S2). GM-CSF (ρ = −0.535, *P* = 0.007), IL-1α (ρ = −0.622, *P* = 0.001), IL-1β (ρ = −0.509, *P* = 0.011), IL-2 (ρ = −0.426, *P* = 0.038), IL-6 (ρ = −0.501, *P* = 0.013), IL-16 (ρ = −0.450, *P* = 0.031) and MCP-1 levels (ρ = −0.533, *P* = 0.013) were inversely correlated with VABS composite score, while MIP-1δ levels were directly correlated with VABS composite score (ρ = 0.475, *P* = 0.019). Almost all the cytokines showed significant correlations with each other (Additional file 3: Table S3). IL-6sR (ρ = −0.502, *P* = 0.040), MIP-1β (ρ = −0.524, *P* = 0.031) and MIP-1δ (ρ = −0.516, *P* = 0.034) levels inversely correlated with SRS total T score. IL-6sR levels displayed significant correlation with MIP-1δ (ρ = 0.418, *P* = 0.038).

BLC (ρ = −0.474, *P* = 0.035) and TIMP-2 (ρ = −0.702, *P* = 0.001) significantly correlated with head circumference.

GM-CSF (ρ = −0.590, *P* = 0.026), IL-1β (ρ = −0.709, *P* = 0.005), IL-6 (ρ = −0.672, *P* = 0.009), IL-7 (ρ = −0.670, *P* = 0.009), IL-11 (ρ = −0.891, *P* = 0.00002), IL-12p70 (ρ = −0.722, *P* = 0.005) IL-13 (ρ = −0.816, *P* = 0.0004), IL-16 (ρ = −0.790, *P* = 0.001), IL-17 (ρ = −0.798, *P* = 0.001), M-CSF (ρ = −0.570, *P* = 0.033), and TNF-sRII (ρ = −0.604, *P* = 0.029) all correlated with full IQ. Almost all the cytokines showed significant correlations with each other (see Additional file 4: Table S4), except for TNF-sRII.

### Cytokine profiles and clinical subgroups

The analyses of cytokine profiles in the four clinical subgroups, 1) children with ASD who were non-verbal, 2) children with ASD presenting with GI issues, 3) children with ASD exhibiting regression, and 4) children with ASD with a history of allergies, were conducted using the Mann–Whitney test (to compare the cytokine levels between children with ASD presenting and those not presenting the clinical variable), and the two paired sample signed Wilcoxon rank sum test (to compare the cytokine levels between discordant sibling pairs according to the clinical subgroup of the subject with ASD).

**Figure 1:**
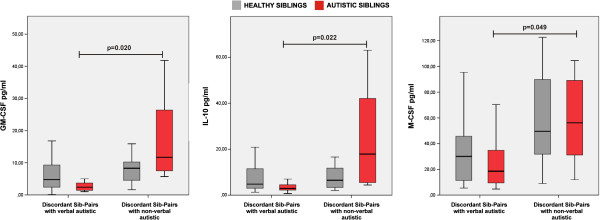
**Cytokines were increased in non-verbal children with autism spectrum disorder (ASD).** Non-verbal children with ASD (n = 7) and their siblings (n = 7), and verbal children with ASD (n = 18) and their siblings (n = 18). The boxes stretch from the 25th to the 75th percentile, the lines across the boxes indicate the median values, and the lines stretching from the boxes indicate extreme values. Statistical significance (*P*<0.05) is reported.

Non-verbal children with ASD displayed higher levels of GM-CSF (*z* = 2.330, *P* = 0.020), IL-10 (*z* = 2.290, *P* = 0.022) and M-CSF (*z* = 1.970, *P* = 0.049) than did verbal children with ASD (Figure 1). Notably, GM-CSF significantly correlated with IL-10 (ρ = 0.782, *P* = 1.062 × 10^-5^) and M-CSF (ρ = 0.585, *P* = 0.002) levels, whereas IL-10 significantly correlated with M-CSF (ρ = 0.565, *P* = 0.005) levels.

**Figure 2:**
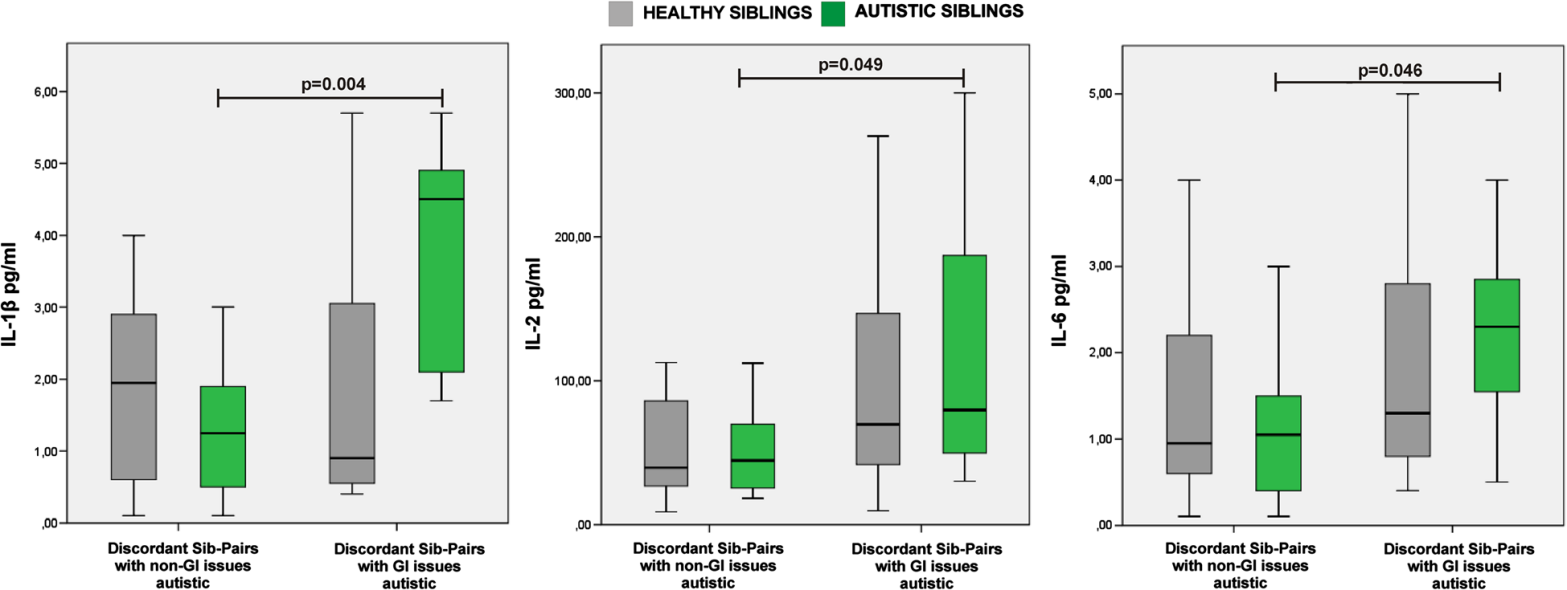
**Cytokine increased in children with autism spectrum disorder (ASD) with gastrointestinal (GI) issues.** We assessed children with ASD with GI issues (n = 7) and their siblings (n = 7), as well as children with ASD with no GI issues (n = 18) and their siblings (n = 18). The boxes stretch from the 25th to the 75th percentile, the lines across the boxes indicate the median values, and the lines stretching from the boxes indicate extreme values. Statistical significance (*P*<0.05) is reported.

Children with ASD with GI issues had higher levels of IL-1β (*z* = 2.870, *P* = 0.004), IL-2 (*z* = 1.970, *P* = 0.049) and IL-6 (*z* = 2.000, *P* = 0.046) than did children with ASD with no GI issues (Figure 2). IL-1β significantly correlated with IL-2 (ρ = 0.455, *P* = 0.022) and IL-6 (ρ = 0.556, *P* = 0.004) levels, and IL-2 significantly correlated with IL-6 (ρ = 0.643, *P* = 0.001) levels.

**Figure 3:**
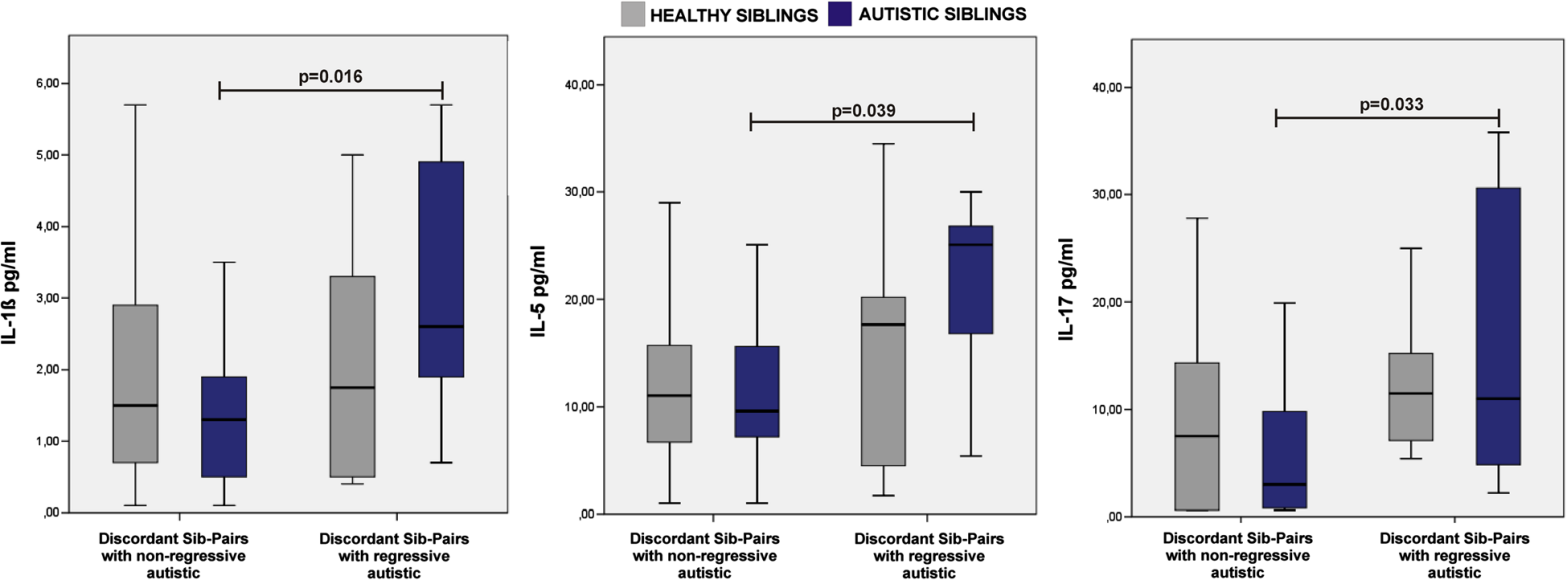
**Cytokine increased in children with autism spectrum disorder (ASD) with regression.** We assessed children with ASD (n = 8) and their siblings (n = 8), as well as children with ASD without regression (n = 17) and their siblings (n = 17). The boxes stretch from the 25th to the 75th percentile, the lines across the boxes indicate the median values, and the lines stretching from the boxes indicate extreme values. Statistical significance (*P*<0.05) is reported.

IL-1β (*z* = 2.420, *P* = 0.016), IL-5 (*z* = 2.060, *P* = 0.039) and IL-17 (*z* = 2.130, *P* = 0.033) levels were higher in children with ASD with regression than in those with no regression (Figure 3). IL-1β significantly correlated with IL-5 (ρ = 0.523, *P* = 0.009) and IL-17 (ρ = 0.497, *P* = 0.012) levels, while IL-5 significantly correlated with IL-17 (ρ = 0.484, *P* = 0.017) levels. Interestingly, levels of Th2 (*z* = 1.990, *P* = 0.047) and Th17 (*z* = 2.040, *P* = 0.041) cytokines were significantly higher in children with regressive ASD than in children with ASD who had no regression. No significant difference was seen in cytokine levels between children with ASD who had a history of allergies and those who had no history of allergies.

## Discussion

Despite the great wealth of data on autism gained to date, and the possible involvement of the immune system in ASD, the results are still inconclusive. This is probably attributable to the large phenotypic and genetic heterogeneity of ASD [37]. In an attempt to identify the complex immune pathogenetic components underlying ASD, we carried out a comprehensive analysis of cytokines and chemokines in discordant sibling pairs. We explored the hypothesis that the use of discordant sibling pairs may reduce heterogeneity and facilitate the identification of immune underpinnings in ASD.

We did not find any significant differences in cytokine levels between children with ASD and their related healthy siblings. This is in line with a previous study by Saresella *et al*. [38] which showed that the immune profiles of children with autism did not differ from their typically developing siblings. That study indicated the presence of an ‘autism endophenotype’ that expands immune dysfunction to family members who are seemingly unaffected by the core symptoms of autism. Moreover, anti-brain antibodies were found both in children with autism and in their unaffected siblings [39]. Notably, healthy siblings are characterized by subtle neurologic impairment [40-43], and there is evidence of atypical social and communication development during infancy [44]. Recent studies also described ASD as the quantitative extreme of a neurodevelopmental continuum in the general population [45], with moderate genetic heritability and a substantial shared twin environmental component [46]. Moreover, the high percentage of macrocephaly we found among the healthy siblings (23.8%) compared with that reported for general population (around 3%) [47] concurs with the evidence of an extended familial endophenotype. Thus, the lack of significant differences between sibling pairs discordant for ASD found in our study is in line with the results of previous studies. It is possible that a common immunogenetic background shared by siblings might eventually lead to different clinical outcomes when an environmental stress (for example, prenatal exposure to environmental toxins, viral and bacterial infections, parental microchimerism, etc.) occurs during development.

**Figure 4:**
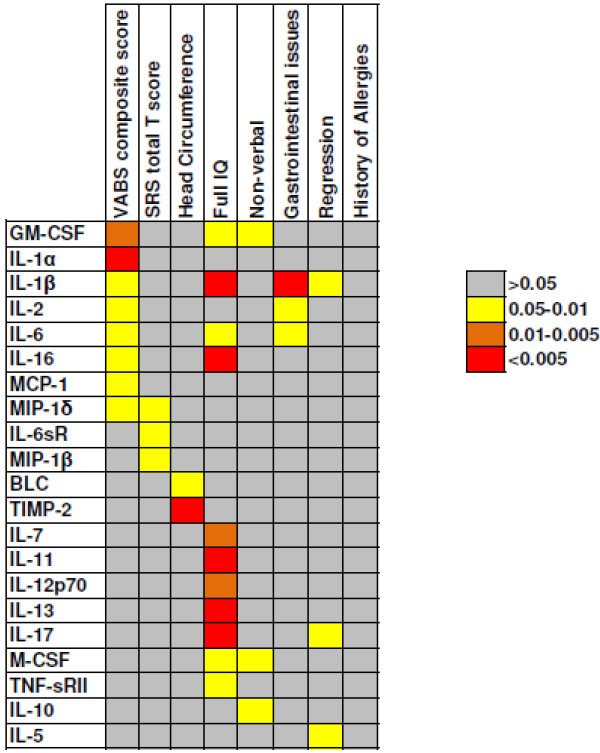
**Summary table of cytokines associated with quantitative traits and clinical subgroups analyzed.***P* value ranges for association/correlation are given.

However, the cytokine/chemokine levels in our subjects did correlate with the quantitative clinical traits, and these were significantly different when the clinical subgroups were analyzed (Figure 4). It is notable that these differences seem to be attributable to a predisposing immunogenetic background, as no other significant differences were noticed between discordant sibling pairs.

IL-1β appears to be the cytokine most involved in the quantitative traits and clinical subgroups of ASD (Figure 4). Involvement of IL-1β in the physiopathology of autism is generally supported by several studies reporting higher levels of this cytokine in the plasma of children with ASD, high-functioning children with ASD, and adults with severe ASD compared with unrelated controls [48]. It has been shown that peripheral blood cells from subjects with ASD produced higher levels of IL-1β both at baseline and after stimulation with Toll-like receptor (TLR)2 or TLR4 [48]. Moreover, plasma levels of IL-1β were found to correlate with regressive onset [20], a finding consistent with the current results. Ashwood *et al*. [20] also showed that increased cytokine levels occurred predominantly in children who had a regressive form of ASD. In line with this, we also found increased levels of IL-1β, IL-5, and IL-17, together with total Th2 and Th17 cytokine levels, in children with regressive ASD compared with children with ASD who had no regression. All these findings are compatible with the established evidence that levels of immunomodulatory factors are altered in children with regressive ASD compared with children with non-regressive ASD [49-52]. It is notable that we found increased levels of IL-5 in children who had a regressive form of ASD. Notably, high levels of IL-5 in mid-gestation maternal serum samples were significantly associated with a 50% increased risk of ASD in the offspring, and especially of regressive autism [53]. Further, an increased level of IL-5 has been reported in male subjects with high-functioning ASD/Asperger’s disorder [17,25]. IL-5 is produced by Th2 cells and mast cells [54]. Its functions are to stimulate B-cell growth and increase immunoglobulin secretion. An IL-5 transgenic mouse model has two predominant features: 1) a marked increase in B-1 cells, resulting in enhanced serum antibody levels, and 2) an expansion of eosinophil numbers in the blood and eosinophil infiltration into various tissues [55]. The presence of maternal auto-antibodies specific for fetal brain proteins has also been shown to be strongly associated with regressive autism [56].

We found the levels of several cytokines (GM-CSF, IL-1α, IL-1β, IL-2, IL-6, IL-16, and MCP-1) to be inversely correlated with the VABS composite score. Increased levels of IL-1β, IL-6, and MCP-1 had previously been shown to be associated with more aberrant behaviors or impairments in cognitive and adaptive function of patients with ASD [20,48,57]. The level of MCP-1 was found to be increased in astrocytes in the anterior cingulate gyrus, and also in the cerebellum and in brain tissue homogenates in patients with ASD [58]. Moreover, a 12-fold increase in MCP-1 was also noted in the cerebrospinal fluid of children with ASD children compared with controls [58]. Notably, it has also been shown that MCP-1 expression is induced by IL-1 [59]. Thus, it is conceivable that increased levels of the two pro-inflammatory cytokines IL-1α and IL-1β might trigger the expression of MCP-1. It should be noted that IL-6 has a crucial role in the development and plasticity of the central nervous system, and it was shown to be significantly increased in the cerebellum of subjects with ASD [60]. Furthermore, IL-6 overexpression in granule cells causes impairments in the adhesion and migration of these cells, possibly altering neural-cell adhesion and migration, and causing an imbalance of excitatory and inhibitory circuits. Moreover, IL-6 exerts a striking negative effect through maternal immune activation on fetal brain development during pregnancy [61]. In addition, a significantly higher level of IL-6 was previously found in the plasma of children with ASD compared with that of typically developing children [20]. Overall, it should be noted that all the cytokines that inversely correlated with the VABS composite score in our study exert a pro-inflammatory function. This finding supports the view that increased levels of pro-inflammatory cytokines can contribute to greater impairments in behavior, characteristic of the core features of ASD, particularly deficits in social interaction and communication [4]. IL-6sR, MIP-1β, and MIP-1δ were inversely correlated with the SRS total score, suggesting a positive beneficial role for higher levels of these cytokines in autism-related social behavior. However, these results should be interpreted with caution, because the role of these cytokines in ASD is still unclear.

We also found a correlation of two cytokine/chemokines (BLC/CXCL13 and TIMP-2) with head circumference, which is the first such report, to our knowledge. Head circumference represents one of the most reliable, consistent, and easily detectable endophenotypes in autism research. Macrocephaly has been consistently recorded in approximately 20% (range 14% to 37%) of patients with autism [36]. Excessive neurite outgrowth and reduced terminal pruning during infancy is believed to play a crucial role in the establishment of macrocephaly [36]. It has been shown that TIMP-2 [62] affects neurite outgrowth, making its strong correlation with head circumference in this study highly plausible. The effect of BLC/CXCL13 on head circumference might result from its interaction with the IL-7 receptor pathway [63], which significantly affects neurite outgrowth [64,65].

We also found associations between plasma levels of several cytokines (GM-CSF, IL-1β, IL-6, IL-7, IL-11, IL-12p70, IL-13, IL-16, IL-17, M-CSF, and TNF-sRII) with full IQ. It is currently unclear how cytokines might affect IQ during childhood in ASD, thus these data should be treated with caution until further validation can be performed.

Language impairment is a common feature of ASD, and some individuals with ASD never acquire language [27]. Studies conducted on the *FOXP2* gene, which is related to the speech-language disorder, unveiled the crucial role of the cerebellum and Purkinje cells in the pathogenesis of speech-language disorders [66]. *Foxp2* (R552H) knock-in mice showed severe ultrasonic vocalization, motor impairment, and immature Purkinje cells with poor dendrites and fewer synapses [66]. We found increased levels of GM-CSF, M-CSF, and IL-10 in non-verbal children with ASD compared with verbal children with ASD. Purkinje cells express receptors for both GM-CSF [67] and M-CSF [68], thus it is possible that altered levels of these two growth factors influence the maturation of the Purkinje cells, leading to language impairment. Notably, IL-10 is known to inhibit the action of GM-CSF [69], and also to act in synergy with M-CSF [70], thus, its involvement in non-verbal ASD, together with that of GM-CSF and M-CSF, seems highly conceivable.

We found that children with ASD with GI issues displayed significantly higher levels of IL-1β, IL-2, and IL-6. The relatively high frequency and variable spectrum of GI symptoms, reported by many parents of autistic children, could conceivably stem from a complex combination of abnormal gut microbiome, excessive intestinal permeability, local immune dysreactivity, and possibly pleiotropic roles of autism genes in nervous and gut tissue [71]. In this context, it should be notes that increased concentrations of IL-1β and IL-2 have been reported in endoscopic mucosal biopsy specimens from patients with inflammatory bowel disease [72]. It has also been shown that IL-1β and IL-2 production is significantly increased in active ulcerative colitis and is significantly correlated to its activity index [73]. Increased level of IL-6 may result from high levels of IL-1β and IL-2, two cytokines that stimulate IL-6 expression in human monocytes by independent mechanisms [74].

Correlation and association do not imply causation. Even assuming that pathophysiological mechanisms do link cytokines to the quantitative traits and clinical subgroups analyzed in this study, the nature of these mechanisms remains open to interpretation. Nonetheless, our results provide important clues pointing toward possible mechanisms, which will deserve closer scrutiny in future investigations.

Whereas previous studies on plasma/serum cytokine profiling in ASD used bead-based suspension arrays [17,20,25,26,57], we used a planar array. Planar microarray comprises reagents for individual tests immobilized as an ordered array or grid of discrete reagent areas (spots) on a flat surface (for example, a microscope slide) [75]. The bead-microarray format comprises encoded microbeads (for example, each with a unique fluorescence signature), and each type of bead is coated with a different reagent [75]. Our planar array is based on sandwich ELISA-based technology, which achieves higher sensitivity and specificity, and is the method of choice for low-abundance proteins [76]. Overall, most studies to date have reported good correlation between bead-based and planar cytokine arrays and between multiplex assays and traditional ELISA (comparable relative changes), thus recommending the continued and expanded use of multiplexing systems in high-throughput screening applications [77].

### Limitations

The present study has several limitations. First, our findings should be viewed as preliminary, owing to our small sample size. Nonetheless, the adoption of strict inclusion criteria for patient enrollment and the careful assessment of clinical variables ensured specificity and should enhance the reproducibility of the present results. Secondly, several cytokines previously found to be associated with ASD, including IL-23, transforming growth factor (TGF)-β1, growth-regulated oncogene-α, and macrophage inhibitory factor), are not measurable by the array-based multiplex assay we used ([4,25]). Further, both IL-23 and TGF-β1 are essential components of the Th17-based immune response. Consequently, our data on Th17 cytokine subset are limited and should be viewed with caution.

## Conclusion

Overall, the present study reports the lack of significant differences in plasma-cytokine levels between children with ASD and in their related non-autistic siblings. Thus, our results support the evidence that the immune profiles of children with autism do not differ from their typically developing siblings [38]. However, the significant association of cytokine levels with the quantitative traits and the clinical subgroups analyzed suggests that altered immune responses may affect core features of ASD [20]. Because ASD may encompass several distinct phenotypes, the potential partition of ASD subgroups based on immunological parameters and/or associations with worsening behavior may have important implications for diagnosis, and for the design and monitoring of therapeutic treatments of ASD [20]. Clearly, further studies are warranted to confirm and extend the associations found in the present study, and should also include analysis of other relevant inflammatory molecules, such as auto-antibodies [9-13].

## Competing interests

The authors have declared that no competing interests exist. Ray Biotech Inc., the commercial funder, had no role in study design, data collection and analysis, decision to publish, or preparation of the manuscript.

## Authors’ contributions

MJH, VN and BO-R conceived and designed the experiments. BO-R, VN, SS, and TP performed the experiments. CJS, RDM, SOR, and JK were involved in clinical data collection. VN, MJH, BO-R, and JJC analyzed the data. VN, MJH, BO-R, CJS, AMP, and DWC wrote the paper. All authors read and approved the final manuscript.

## Supplementary Material

Additional file 1: Table S1 Cytokine levels in children with autism spectrum disorder (ASD) and their related healthy siblings. Data are expressed as median (interquartile range). Data analysis was performed using the non-parametric two paired samples signed-rank test (Wilcoxon). *Z* scores and *P* values are reported. Click here for file

Additional file 2: Table S2 Correlation analyses between cytokine levels and quantitative clinical traits. Data analysis was performed by non-parametric were performed by Spearman’s rank correlation analysis (ρ). *R* and *P* values are reported. Significant results are highlighted in bold. Click here for file

Additional file 3: Table S3 Analysis of correlations between cytokines associated with VABS composite score. Data analysis was performed by non-parametric were performed by Spearman’s rank correlation analysis (ρ). *R* and *P* values are reported. Significant results are highlighted in bold. Click here for file

Additional file 4: Table S4. Correlation analysis of cytokines associated with full intelligent quotient. Data analysis was performed by non-parametric were performed by Spearman’s rank correlation analysis (ρ). *R* and *P* values are reported. Significant results are highlighted in bold. Click here for file
