# Supplementary material for: Plasma cytokine profiling in sibling pairs discordant for autism spectrum disorder
Source: J Neuroinflammation. 2013 Mar 14;10:38. doi: 10.1186/1742-2094-10-38 (PMC3616926; doi:10.1186/1742-2094-10-38)
Supplement: Additional file 1: Table S1 — Cytokine levels in children with autism spectrum disorder (ASD) and their related healthy siblings. Data are expressed as median (interquartile range). Data analysis was performed using the non-parametric two paired samples signed-rank test (Wilcoxon). Z scores and P values are reported. [file 1742-2094-10-38-S1.doc]

**Table S1. Cytokine levels in autistics and their related healthy siblings.** Data are expressed as median and (Interquartile ranges). Data analysis was performed by non-parametric Two Paired Sample Signed Rank Test (Wilcoxon). Z-scores and p-values are reported.

|  | Autistics | Healthy Siblings | z-score | p-value |
| --- | --- | --- | --- | --- |
| **BLC** | 280.5 (180.9-365.6) | 294.3 (157.7-421.1) | -0.067 | 0.946 |
| **Eotaxin** | 43.2 (17.4-66.8) | 40.3 (26.7-51.5) | 0.329 | 0.742 |
| **Eotaxin-2** | 152.5 (65.0-333.5) | 152.6 (113.7-310.6) | 0.040 | 0.968 |
| **G-CSF** | 181.9 (129.2-275.7) | 194.1 (134.1-253.1) | 0.632 | 0.527 |
| **GM-CSF** | 4.2 (1.9-13.5) | 6.2 (3.0-10.3) | 0.229 | 0.819 |
| **I-309** | 63.2 (28.1-146.6) | 42.3 (22.2-101.6) | 0.314 | 0.753 |
| **ICAM-1** | 64702.5 (50257.4-93271.1) | 61800.1 (33631.9-108913.5) | 0.229 | 0.819 |
| **IFN-γ** | 11.0 (6.5-18.8) | 7.9 (5.0-13.8) | 0.874 | 0.382 |
| **IL-1α** | 14.1 (8.6-28.8) | 14.9 (5.2-21.3) | 0.578 | 0.563 |
| **IL-1β** | 1.7 (0.7-2.3) | 1.5 (0.6-2.9) | -0.314 | 0.753 |
| **IL-1ra** | 50.8 (30.0-91.2) | 50.4 (32.5-60.2) | 0.632 | 0.527 |
| **IL-2** | 48.5 (27.7-78.2) | 51.6 (26.7-86.8) | -0.417 | 0.677 |
| **IL-4** | 8.6 (4.9-28.8) | 8.8 (5.4-29.9) | -1.157 | 0.247 |
| **IL-5** | 14.2 (7.4-23.7) | 11.5 (7.6-18.4) | 0.943 | 0.346 |
| **IL-6** | 1.4 (0.5-2.2) | 1.0 (0.7-2.3) | 0.202 | 0.840 |
| **IL-6sR** | 396.5 (343.8-625.0) | 454.6 (361.8-645.1) | -0.971 | 0.331 |
| **IL-7** | 5.9 (2.6-9.8) | 6.1 (3.4-8.9) | 0.429 | 0.668 |
| **IL-8** | 2.0 (1.1-3.7) | 2.5 (1.0-3.1) | -0.030 | 0.976 |
| **IL-10** | 4.4 (2.4-18.7) | 4.8 (2.9-13.0) | 0.400 | 0.689 |
| **IL-11** | 19.8 (10.0-56.5) | 22.2 (10.9-42.1) | 0.000 | 1.000 |
| **IL-12p40** | 87.5 (49.4-163.7) | 109.0 (43.6-260.1) | -0.486 | 0.627 |
| **IL-12p70** | 0.8 (0.2-2.2) | 0.6 (0.2-3.7) | -0.896 | 0.370 |
| **IL-13** | 1.1 (0.7-3.9) | 2.5 (0.8-3.8) | -0.715 | 0.475 |
| **IL-15** | 57.9 (33.1-97.1) | 50.5 (23.3-71.4) | 0.000 | 1.000 |
| **IL-16** | 38.6 (15.9-254.9) | 39.7 (11.8-366.4) | -0.601 | 0.548 |
| **IL-17** | 5.2 (1.5-10.6) | 8.1 (2.4-14.3) | -0.563 | 0.574 |
| **MCP-1** | 132.8 (105.5-177.9) | 113.3 (98.7-168.4) | 0.713 | 0.476 |
| **M-CSF** | 25.9 (13.6-63.3) | 37.8 (11.4-87.5) | -0.971 | 0.331 |
| **MIG** | 283.6 (159.6-381.7) | 308.2 (194.4-399.3) | -0.700 | 0.484 |
| **MIP-1α** | 70.3 (27.3-133.0) | 68.6 (22.8-148.3) | 0.043 | 0.966 |
| **MIP-1β** | 54.1 (39.8-82.4) | 53.6 (38.2-102.4) | -0.471 | 0.638 |
| **MIP-1δ** | 2091.6 (684.7-3172.8) | 2069.3 (1090.8-3091.3) | 0.417 | 0.677 |
| **PDGF-BB** | 918.9 (674.8-1267.3) | 913.9 (721.7-1362.5) | -0.471 | 0.638 |
| **RANTES** | 5308.7 (4765.9-6244.7) | 5626.5 (4915.0-7473.5) | -0.767 | 0.443 |
| **TIMP-1** | 27083.9 (16565.2-54766.7) | 25535.5 (18105.9-43174.6) | 0.686 | 0.493 |
| **TIMP-2** | 43888.2 (25222.2-72111.9) | 46628.1 (33618.3-69997.7) | -0.363 | 0.716 |
| **TNF-α** | 25.2 (13.3-37.2) | 27.2 (13.7-37.0) | -0.429 | 0.668 |
| **TNF-β** | 15.2 (6.1-37.4) | 15.7 (6.1-33.7) | 0.091 | 0.927 |
| **TNF sRI** | 1209.8 (785.3-1982.1) | 1028.4 (887.1-1556.9) | 0.740 | 0.459 |
| **TNF sRII** | 2195.7 (1584.8-3048.0) | 2196.4 (1524.9-3311.9) | 0.274 | 0.784 |
