# Supplementary material for: Plasma cytokine profiling in sibling pairs discordant for autism spectrum disorder
Source: J Neuroinflammation. 2013 Mar 14;10:38. doi: 10.1186/1742-2094-10-38 (PMC3616926; doi:10.1186/1742-2094-10-38)
Supplement: Additional file 2: Table S2 — Correlation analyses between cytokine levels and quantitative clinical traits. Data analysis was performed by non-parametric were performed by Spearman’s rank correlation analysis (ρ). R and P values are reported. Significant results are highlighted in bold. [file 1742-2094-10-38-S2.doc]

**Table S2. Correlation analyses between cytokine levels and quantitative clinical traits.** Data analysis was performed by non-parametric were performed by Spearman’s rank correlations (rho). R-values and p-values are reported. Significant results are highlighted in bold.

|  | **VABS composite score** | | **SRS T score** | | **Head Circumference** | | **Full IQ** | |
| --- | --- | --- | --- | --- | --- | --- | --- | --- |
|  | *ρ* | *p-value* | *ρ* | *p-value* | *ρ* | *p-value* | *ρ* | *p-value* |
| **BLC** | -0.231 | 0.276 | -0.120 | 0.646 | **-0.474** | **0.035** | -0.504 | 0.066 |
| **Eotaxin** | 0.065 | 0.762 | -0.337 | 0.186 | -0.152 | 0.524 | 0.084 | 0.776 |
| **Eotaxin-2** | -0.389 | 0.061 | 0.233 | 0.368 | -0.245 | 0.297 | -0.438 | 0.117 |
| **G-CSF** | 0.220 | 0.301 | -0.361 | 0.155 | -0.094 | 0.694 | -0.029 | 0.923 |
| **GM-CSF** | **-0.535** | **0.007** | 0.306 | 0.233 | 0.156 | 0.511 | **-0.590** | **0.026** |
| **I-309** | -0.050 | 0.818 | -0.320 | 0.210 | -0.268 | 0.253 | -0.394 | 0.164 |
| **ICAM-1** | 0.033 | 0.880 | -0.286 | 0.266 | 0.064 | 0.790 | 0.158 | 0.589 |
| **IFN-γ** | -0.361 | 0.083 | 0.119 | 0.649 | -0.273 | 0.244 | -0.279 | 0.333 |
| **IL-1α** | **-0.622** | **0.001** | 0.476 | 0.054 | 0.055 | 0.819 | -0.136 | 0.642 |
| **IL-1β** | **-0.509** | **0.011** | 0.236 | 0.362 | -0.108 | 0.652 | **-0.709** | **0.005** |
| **IL-1ra** | -0.028 | 0.896 | -0.200 | 0.442 | 0.036 | 0.879 | -0.194 | 0.507 |
| **IL-2** | **-0.426** | **0.038** | 0.307 | 0.231 | -0.306 | 0.189 | -0.163 | 0.578 |
| **IL-4** | -0.328 | 0.118 | 0.028 | 0.914 | 0.139 | 0.560 | -0.307 | 0.285 |
| **IL-5** | -0.349 | 0.102 | 0.165 | 0.542 | -0.229 | 0.345 | -0.500 | 0.069 |
| **IL-6** | **-0.501** | **0.013** | 0.115 | 0.659 | -0.225 | 0.341 | **-0.672** | **0.009** |
| **IL-6sR** | 0.223 | 0.295 | **-0.502** | **0.040** | 0.198 | 0.402 | -0.194 | 0.507 |
| **IL-7** | -0.264 | 0.212 | 0.109 | 0.676 | -0.271 | 0.248 | **-0.670** | **0.009** |
| **IL-8** | 0.029 | 0.895 | -0.338 | 0.185 | 0.064 | 0.789 | -0.367 | 0.196 |
| **IL-10** | -0.347 | 0.114 | 0.191 | 0.494 | 0.241 | 0.335 | -0.533 | 0.061 |
| **IL-11** | -0.326 | 0.120 | 0.139 | 0.596 | -0.148 | 0.532 | **-0.891** | **0.00002** |
| **IL-12p40** | -0.105 | 0.626 | -0.031 | 0.905 | -0.236 | 0.316 | -0.418 | 0.137 |
| **IL-12p70** | -0.365 | 0.095 | 0.287 | 0.299 | -0.077 | 0.762 | **-0.722** | **0.005** |
| **IL-13** | -0.381 | 0.073 | 0.130 | 0.631 | -0.171 | 0.484 | **-0.816** | **0.0004** |
| **IL-15** | -0.362 | 0.082 | 0.032 | 0.903 | -0.097 | 0.684 | -0.251 | 0.387 |
| **IL-16** | **-0.450** | **0.031** | 0.152 | 0.560 | -0.073 | 0.773 | **-0.790** | **0.001** |
| **IL-17** | -0.289 | 0.171 | 0.193 | 0.458 | -0.120 | 0.615 | **-0.798** | **0.001** |
| **MCP-1** | **-0.533** | **0.013** | 0.385 | 0.175 | 0.030 | 0.905 | -0.490 | 0.106 |
| **M-CSF** | -0.313 | 0.137 | -0.090 | 0.733 | -0.162 | 0.495 | **-0.570** | **0.033** |
| **MIG** | -0.086 | 0.698 | -0.116 | 0.656 | 0.044 | 0.854 | -0.019 | 0.950 |
| **MIP-1α** | -0.262 | 0.216 | -0.178 | 0.495 | -0.006 | 0.980 | -0.497 | 0.070 |
| **MIP-1β** | 0.160 | 0.456 | **-0.524** | **0.031** | -0.224 | 0.342 | -0.400 | 0.156 |
| **MIP-1δ** | **0.475** | **0.019** | **-0.516** | **0.034** | 0.164 | 0.491 | -0.169 | 0.563 |
| **PDGF-BB** | 0.259 | 0.221 | -0.113 | 0.666 | -0.082 | 0.732 | -0.092 | 0.753 |
| **RANTES** | 0.243 | 0.252 | -0.429 | 0.086 | 0.102 | 0.670 | -0.339 | 0.236 |
| **TIMP-1** | -0.198 | 0.354 | 0.050 | 0.848 | -0.047 | 0.844 | -0.574 | 0.032 |
| **TIMP-2** | -0.139 | 0.518 | -0.148 | 0.570 | **-0.702** | **0.001** | -0.304 | 0.291 |
| **TNF-α** | -0.277 | 0.189 | 0.110 | 0.673 | 0.052 | 0.827 | -0.360 | 0.206 |
| **TNF-β** | -0.376 | 0.070 | 0.257 | 0.319 | -0.007 | 0.977 | -0.356 | 0.211 |
| **TNF sRI** | 0.050 | 0.816 | -0.201 | 0.439 | 0.086 | 0.717 | 0.128 | 0.664 |
| **TNF sRII** | -0.365 | 0.087 | 0.259 | 0.333 | -0.154 | 0.529 | **-0.604** | **0.029** |
