# Supplementary material for: Plasma cytokine profiling in sibling pairs discordant for autism spectrum disorder
Source: J Neuroinflammation. 2013 Mar 14;10:38. doi: 10.1186/1742-2094-10-38 (PMC3616926; doi:10.1186/1742-2094-10-38)
Supplement: Additional file 3: Table S3 — Analysis of correlations between cytokines associated with VABS composite score. Data analysis was performed by non-parametric were performed by Spearman’s rank correlation analysis (ρ). R and P values are reported. Significant results are highlighted in bold. [file 1742-2094-10-38-S3.doc]

**Table S3. Correlation analysis among cytokines associated with VABS composite score.** Data analysis was performed by non-parametric were performed by Spearman’s rank correlations (ρ). R-values and p-values are reported. Significant results are highlighted in bold.

|  | | **GM-CSF** | **IL-1α** | **IL-1β** | **IL-2** | **IL-6** | **IL-16** | **MCP-1** | **MIP-1δ** |
| --- | --- | --- | --- | --- | --- | --- | --- | --- | --- |
| **GM-CSF** | ρ |  | **0.505** | **0.661** | 0.334 | **0.611** | **0.630** | **0.561** | -0.230 |
|  | p-value |  | **0.010** | **3.2x10^-4^** | 0.102 | **0.001** | **0.001** | **0.007** | 0.268 |
| **IL-1α** | ρ | **0.505** |  | **0.568** | **0.682** | **0.548** | **0.435** | 0.248 | **-0.445** |
|  | p-value | **0.010** |  | **0.003** | **1.7x10^-4^** | **0.005** | **0.038** | 0.266 | **0.026** |
| **IL-1β** | ρ | **0.661** | **0.568** |  | **0.455** | **0.556** | **0.618** | 0.335 | -0.063 |
|  | p-value | **3.2x10^-4^** | **0.003** |  | **0.022** | **0.004** | **0.002** | 0.127 | 0.766 |
| **IL-2** | ρ | 0.334 | **0.682** | **0.455** |  | **0.643** | 0.371 | 0.142 | **-0.483** |
|  | p-value | 0.102 | **1.7x10^-4^** | **0.022** |  | **0.001** | 0.081 | 0.529 | **0.014** |
| **IL-6** | ρ | **0.611** | **0.548** | **0.556** | **0.643** |  | **0.475** | 0.373 | -0.162 |
|  | p-value | **0.001** | **0.005** | **0.004** | **0.001** |  | **0.022** | 0.087 | 0.440 |
| **IL-16** | ρ | **0.630** | **0.435** | **0.618** | 0.371 | **0.475** |  | **0.611** | **-0.470** |
|  | p-value | **0.001** | **0.038** | **0.002** | 0.081 | **0.022** |  | **0.004** | **0.024** |
| **MCP-1** | ρ | **0.561** | 0.248 | 0.335 | 0.142 | 0.373 | **0.611** |  | -0.139 |
|  | p-value | **0.007** | 0.266 | 0.127 | 0.529 | 0.087 | **0.004** |  | 0.536 |
| **MIP-1δ** | ρ | -0.230 | **-0.445** | -0.063 | **-0.483** | -0.162 | **-0.470** | -0.139 |  |
|  | p-value | 0.268 | **0.026** | 0.766 | **0.014** | 0.440 | **0.024** | 0.536 |  |
