# Supplementary material for: Plasma cytokine profiling in sibling pairs discordant for autism spectrum disorder
Source: J Neuroinflammation. 2013 Mar 14;10:38. doi: 10.1186/1742-2094-10-38 (PMC3616926; doi:10.1186/1742-2094-10-38)
Supplement: Additional file 4: Table S4. — Correlation analysis of cytokines associated with full intelligent quotient. Data analysis was performed by non-parametric were performed by Spearman’s rank correlation analysis (ρ). R and P values are reported. Significant results are highlighted in bold. [file 1742-2094-10-38-S4.doc]

**Table S4. Correlation analysis among cytokines associated with full IQ.** Data analysis was performed by non-parametric were performed by Spearman’s rank correlations (rho). R-values and p-values are reported. Significant results are highlighted in bold.

|  | | **GM-CSF** | **IL-1β** | **IL-6** | **IL-7** | **IL-11** | **IL-12p70** | **IL-13** | **IL-16** | **IL-17** | **M-CSF** | **TNF sRII** |
| --- | --- | --- | --- | --- | --- | --- | --- | --- | --- | --- | --- | --- |
| **GM-CSF** | ρ |  | **0.661** | **0.611** | **0.527** | **0.608** | **0.650** | **0.672** | **0.630** | **0.476** | **0.585** | 0.227 |
|  | p-value |  | **3.2x10^-4^** | **0.001** | **0.007** | **0.001** | **0.001** | **3.2x10^-4^** | **0.001** | **0.016** | **0.002** | 0.287 |
| **IL-1β** | ρ | **0.661** |  | **0.556** | 0.340 | **0.486** | **0.585** | **0.518** | **0.618** | **0.497** | **0.668** | 0.295 |
|  | p-value | **3.2x10^-4^** |  | **0.004** | 0.096 | **0.014** | **0.003** | **0.010** | **0.002** | **0.012** | **2.6x10^-4^** | 0.162 |
| **IL-6** | ρ | **0.611** | **0.556** |  | **0.790** | **0.692** | **0.415** | **0.820** | **0.475** | **0.596** | **0.493** | 0.398 |
|  | p-value | **0.001** | **0.004** |  | **2.7x10^-6^** | **1.3x10^-4^** | **0.049** | **9.5x10^-7^** | **0.022** | **0.002** | **0.012** | 0.054 |
| **IL-7** | ρ | **0.527** | 0.340 | **0.790** |  | **0.595** | **0.526** | **0.838** | 0.354 | **0.568** | 0.338 | 0.295 |
|  | p-value | **0.007** | 0.096 | **2.7x10^-6^** |  | **0.002** | **0.010** | **3.2x10^-7^** | 0.097 | **0.003** | 0.099 | 0.161 |
| **IL-11** | ρ | **0.608** | **0.486** | **0.692** | **0.595** |  | **0.563** | **0.660** | **0.651** | **0.901** | **0.569** | **0.441** |
|  | p-value | **0.001** | **0.014** | **1.3x10^-4^** | **0.002** |  | **0.005** | **4.5x10^-4^** | **0.001** | **8.4x10^-10^** | **0.003** | **0.031** |
| **IL-12p70** | ρ | **0.650** | **0.585** | **0.415** | **0.526** | **0.563** |  | **0.624** | **0.847** | **0.534** | 0.343 | 0.317 |
|  | p-value | **0.001** | **0.003** | **0.049** | **0.010** | **0.005** |  | **0.002** | **1.3x10^-6^** | **0.009** | 0.109 | 0.150 |
| **IL-13** | ρ | **0.672** | **0.518** | **0.820** | **0.838** | **0.660** | **0.624** |  | **0.461** | **0.660** | **0.492** | 0.351 |
|  | p-value | **3.2x10^-4^** | **0.010** | **9.5x10^-7^** | **3.2x10^-7^** | **4.5x10^-4^** | **0.002** |  | **0.031** | **4.5x10^-4^** | **0.015** | 0.100 |
| **IL-16** | ρ | **0.630** | **0.618** | **0.475** | 0.354 | **0.651** | **0.847** | **0.461** |  | **0.584** | **0.462** | 0.404 |
|  | p-value | **0.001** | **0.002** | **0.022** | 0.097 | **0.001** | **1.3x10^-6^** | **0.031** |  | **0.003** | **0.027** | 0.062 |
| **IL-17** | ρ | **0.476** | **0.497** | **0.596** | **0.568** | **0.901** | **0.534** | **0.660** | **0.584** |  | **0.457** | **0.489** |
|  | p-value | **0.016** | **0.012** | **0.002** | **0.003** | **8.4x10^-10^** | **0.009** | **4.5x10^-4^** | **0.003** |  | **0.022** | **0.015** |
| **M-CSF** | ρ | **0.585** | **0.668** | **0.493** | 0.338 | **0.569** | 0.343 | **0.492** | **0.462** | **0.457** |  | 0.137 |
|  | p-value | **0.002** | **2.6x10^-4^** | **0.012** | 0.099 | **0.003** | 0.109 | **0.015** | **0.027** | **0.022** |  | 0.522 |
| **TNF sRII** | ρ | 0.227 | 0.295 | 0.398 | 0.295 | **0.441** | 0.317 | 0.351 | 0.404 | **0.489** | 0.137 |  |
|  | p-value | 0.287 | 0.162 | 0.054 | 0.161 | **0.031** | 0.150 | 0.100 | 0.062 | **0.015** | 0.522 |  |
